# A Case of the Utility of Viscoelastic Testing for the Management of the Impella Device in Heparin Anticoagulation

**DOI:** 10.7759/cureus.77917

**Published:** 2025-01-24

**Authors:** Satoshi Kometani, Eri Watanabe, Tomohide Takei, Mimiko Tabata

**Affiliations:** 1 Anesthesiology, Yamato Seiwa Hospital, Yamato, JPN; 2 Cardiovascular Surgery, Yamato Seiwa Hospital, Yamato, JPN

**Keywords:** activated clotting time, heparin administration, impella device, point-of-care viscoelastic testing, unfractionated heparin (ufh)

## Abstract

The main anticoagulant therapy with Impella is unfractionated heparin (UFH), with manufacturers recommending a target therapeutic range of 160-180 seconds for activated clotting time (ACT). However, the ACT assay does not have uniform criteria, and many available devices contain a variety of activators, making it difficult to compare them individually. We used Impella CP in the perioperative management of a post-myocardial infarction ventricular septal rupture in a 69-year-old man. A significant amount of UHF was used in this case, but as the ACT did not reach the recommended range, blood viscoelasticity testing (VET) was adopted as an alternative point-of-care test (POCT). The initial measurement results showed a clot time (CT) of 308 seconds, a heparinase clot time (CTH) of 161 seconds, and a clot time ratio (CTR) of 1.9, indicating that UFH was sufficiently active. Continuous administration of UFH was then performed with reference to VET, which was terminated three hours before surgery. As an alternative POCT to ACT, the VET may be suitable for assessing low doses of UFH effectiveness with heparinase assay, which provides specific measures of the heparin effect, such as CTR, and minimizes the variability in results as compared to ACT.

## Introduction

Among the various mechanical circulatory support (MCS) devices, Impella (Abiomed Inc., Danvers, MA, USA) is a small axial flow pump that can be inserted percutaneously and provides less invasive left ventricular assistance. Impella requires anticoagulation to prevent thrombosis and optimize pump function, and the manufacturer recommends monitoring and managing the activated clotting time (ACT) to within 160-180 seconds [1]. While this is mainly achieved by unfractionated heparin (UFH) administration, there is no guidance on adjusting it. In addition, while various ACT measuring devices are available from different companies, the same blood samples give different results, as the measurement principles (magnet and LED) and activator components (kaolin, celite, glass beads, silica, and phospholipids) are not standardized, and their correlation coefficients are usually 0.7 to 0.9 [2]. These backgrounds, in the absence of clear guidance, may result in over- or under-anticoagulation and increase the potential risk of major cardiac and cerebral events, as well as other vascular, thromboembolic, and hemorrhagic events.

Viscoelastic testing (VET) measures the coagulation reaction and the heparin effect in approximately 10 min by adding kaolin and kaolin + heparinase to the citric acid-added blood sample, which may be helpful as an alternative point-of-care test (POCT). However, to our knowledge, VET is mainly used during the perioperative period of cardiac surgery with a cardiopulmonary bypass, and no reports have been referenced using Impella, so the evaluation thereof has not been established.

We used a transfemoral delivery of Impella CP for perioperative management of severe left heart failure associated with post-myocardial infarction ventricular septal rupture (post-MI VSR). In this case, the target ACT was not reached even after an adequate dose of UFH administration, so anticoagulation therapy was performed with reference to VET. We report this experience and assess its usefulness based on a literature review.

## Case presentation

A 69-year-old male (height 171 cm, weight 98 kg, body surface area 2.1 m^2^) presented to the referring hospital with shortness of breath that appeared a week prior and then worsened. Chest X-ray showed pulmonary congestion and pleural effusion, and electrocardiography demonstrated ST elevation accompanied by Q waves in leads II, III, and aVF. Heart failure associated with subacute inferior wall myocardial infarction was suspected, which led to hospitalization. However, on subsequent transthoracic echocardiography, a left-to-right shunt flow was observed in the septum along with a decrease in wall movement of the left ventricular inferior wall; therefore, the patient was transported to our hospital for emergency treatment with a diagnosis of post-MI VSR. The patient's medical history included no cardiovascular disease risk factors such as smoking, diabetes, hypercholesterolemia, hypertension, or any current medications. The patient's family history was unremarkable.

Upon arrival at our hospital, his blood pressure was 96/73 mmHg, heart rate was 101 bpm, oxygen saturation (SpO_2_) was 97% (O_2_ 3 L/min via face mask), respiratory rate was 20/min, and consciousness level was 15 on the Glasgow Coma Scale. The troponin T qualitative rapid detection kit yielded positive results, and the CK-MB level was within the reference value. Coronary angiography showed complete occlusion of the distal right coronary artery. Coronary artery bypass grafting and ventricular septal defect repair were scheduled, and the Impella CP transfemoral delivery was introduced for left ventricular unloading.

The Impella was inserted percutaneously through the right femoral artery and protected with a purge solution using a 5% glucose solution. Purge flow and pressure were controlled at 13-15 mL/h and 400-500 mmHg, respectively. Pump flow was maintained at 3.3-3.5 L/min (flow level P9, 46,000 rpm). Anticoagulant therapy was consistently administered systemically with a UFH 12,000 U bolus followed by a purge solution (UFH 10 U/mL). According to the manufacturer's recommendations, the therapeutic anticoagulation target was set at an ACT of 160-180 seconds, combined with activated partial thromboplastin time (APTT) measurements every 12-24 hours.

**Figure 1:**
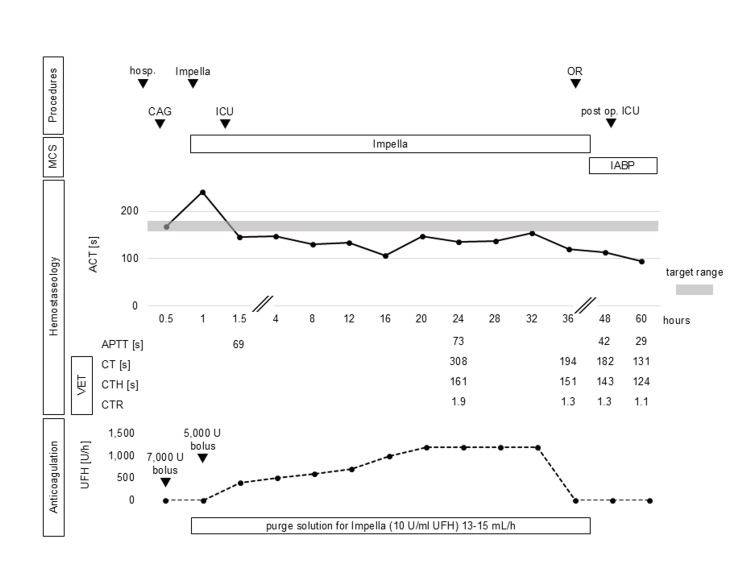
Perioperative time course regarding Impella management and heparin anticoagulation ACT, APTT, CT, CTH, and CTR reference ranges are 97-133 s, 25-40 s, 109-150 s, 113-164 s, and 0.5-1.4, respectively. CAG: coronary angiography, ICU: intensive care unit, OR: operating room, MCS: mechanical circulatory support, IABP: intra-aortic balloon pump, ACT: activated clotting time, VET: viscoelastic testing, APTT: activated partial thromboplastin time, CT: clot time, CTH: heparinase clot time, CTR: clot time ratio, UFH: unfractionated heparin

The ACT measurement device was an Actalyke Mini II (Helena Labs, Beaumont, TX), and the reagent tubes were MAX-ACT (Helena Labs). The ICU time course is shown in Figure 1. The ACT immediately after ICU admission was 145 seconds, indicating inadequate heparinisation. On the other hand, the APTT was 69 seconds, which was discordant and indicated adequate anticoagulation. Antithrombin III activity was within 77% of the reference value, also supporting the expected anticoagulant effect of UFH. However, because the recommended ACT target range was not reached, continuous systemic administration of UFH was started at 400 U/h. UFH was gradually increased to reach the target range, but even under UFH 1,200 U/h, the ACT was 154 seconds, which was not sufficiently prolonged. Since the APTT measured at the same time was prolonged to 73 seconds, we decided not to increase the UFH dose further, considering the risk of bleeding during the following open-heart surgery.

In these circumstances, other anticoagulation monitoring was needed instead of ACT. However, anti-Xa activity was not available for in-hospital measurements, and frequent APTT measurements were impractical because of the human resource status of our institution's laboratory, so VET was performed as an alternative POCT. The Quantra Hemostasis System (Hemosonics, Charlottesville, VA, USA) was used for the VET measurements. The VET results with reference range are shown in Table 1. The results of the initial measurement were a CT of 308 seconds, CTH of 161 seconds, and CTR of 1.9. As with APTT, sufficient prolongation of the CT and CTR was observed. Thereafter, continuous UFH administration was maintained at 1,200 U/h, and the administration was terminated 3 hours before surgery. Preoperative hemostasis was 120 seconds for ACT, 194 seconds for CT, 151 seconds for CTH, and 1.3 for CTR. The surgery was performed under cardiopulmonary bypass with VSR closure (double patch repair) and coronary artery bypass grafting (saphenous vein graft to the right coronary artery). After cardiopulmonary bypass withdrawal, the MCS was switched from Impella to IABP, and surgery was completed. No worsening of heart failure, bleeding, or complications were observed in the postoperative ICU; therefore, the IABP was withdrawn on postoperative day (POD) 2. The hemostasis at this point was ACT 94 seconds, APTT 29 seconds, CT 131 seconds, CTH 124 seconds, and CTR 1.1. Thereafter, the patient was removed from the ventilator on POD 5 and left the ICU on POD 9.

**Table 1:** Viscoelastic testing results with reference range (ICU admission, 0 hours) The ACT indicated inadequate heparinisation. The APTT result was discordant, indicating adequate anticoagulation. (VET adaptation, 24 hours) The initial results showed that UFH was sufficiently active, as with APTT. (3 hours before surgery, 36 hours) UFH was terminated. ACT: activated clotting time, APTT: activated partial thromboplastin time, VET: viscoelastic testing

Parameter	Reference range	0 h	24 h	36 h	48 h	60 h
Clot time (CT)	109 - 150 s		308	194	182	131
Heparinase clot time (CTH)	113 - 164 s		161	151	143	124
Clot time ratio (CTR)	0.5 - 1.4		1.9	1.3	1.3	1.1
ACT (Actalyke Mini)	97 - 133 s	145	136	120	113	94
APTT	25 - 40 s	69	73		42	29

## Discussion

With the spread of primary percutaneous coronary intervention for acute myocardial infarction, post-MI VSRs mechanical complications have decreased, with an incidence rate of approximately 0.3% in recent years [3]. Although the gold standard for post-MI VSR treatment is surgical repair, the mortality rate remains high. This is due to the technical complexity of repairing an infarcted, unclear, and fragile myocardium, coupled with the difficulty of perioperative management of heart failure. In such cases, MCS is known to improve hemodynamics and peripheral organ function, and by performing elective surgery after waiting for the infarct scarring to complete, the prognosis can be improved compared with emergency surgery. Impella is used for left ventricular unloading and as a bridge to VSR closure [4,5].

Knowledge of Impella's anticoagulation management is currently limited, and a standardized approach is needed. At present, setting the UFH infusion rate at approximately 11-12 U/kg/h (not to exceed a maximum of 1,800 U/h) and measuring APTT (target 40-60 seconds) and anti-Xa activity (target 0.3-0.5 U/ml) every four to six hours in combination with ACT is considered standard and ideal; however, consideration thereof must depend on local medical resources and individual cases [6]. In the present case, despite using a considerable amount of UFH, the APTT was confirmed to be sufficiently prolonged but did not reach the target ACT range. As a result, we decided not to increase the UFH dose further, considering the risk of over-anticoagulation. As an alternative anticoagulation monitoring, VET was adopted as a POCT that can be performed only by ICU staff without the laboratory's effort.

The Quantra Hemostasis System, a VET, uses Sonic Estimation of Elasticity via Resonance (SEER) sonoremetry to monitor the coagulation process in cartridge-based microfluidics to measure the shear modulus. The CT and CTH measured in the process measured the coagulation reaction and the evaluation of the heparin effect in approximately 10 min by adding kaolin and kaolin + heparinase to the citric acid-added blood sample, respectively [7,8]. CT and CTR were prolonged in our case and were as helpful as POCT in determining the effect of heparin instead of ACT. For other MCS devices, the literature on VET use during extracorporeal membrane oxygenation (ECMO) management is increasing, and it is noted in the Extracorporeal Life Support Organization (ELSO) guidelines that the heparinase assay can estimate the anticoagulant effect of UFH from the CTR by comparing heparinase-added blood samples with those not added [9]. Therefore, VET under Impella management may be as useful as ECMO. However, to our knowledge, there are no reports in the literature, and an evaluation thereof has not yet been determined.

The reagent tubes used in our case were MAX-ACT tubes. The MAX-ACT assay was developed to use three kinds of coagulation active agents, kaolin, celite, and glass beads, to maximize the conversion from factor XII to factor XIIa and to more accurately and reproducibly correlate with heparin levels than in the past. However, previous studies have also shown that the reliability of ACT to estimate the efficacy of UFH is not always high [2]. It has been reported that MAX-ACT is less susceptible to anticoagulants through more appropriate activation. In on-pump coronary artery bypass surgeries with UFH, MAX-ACT was reported to have consistently lower values than celite-ACT (maximum mean difference 13%) [10]. This phenomenon is a significant problem in lower target ranges and alternative anticoagulation strategies with direct thrombin inhibitors such as direct oral anticoagulants and argatroban. In a study using bivalirudin for off-pump coronary artery bypass surgeries, MAX-ACT was reported to have even lower values than kaolin-ACT and reported results below the therapeutic range (maximum mean difference 27%, maximum mean reduction from 346 to 252 seconds) [11]. In our case, the target ACT was not reached even after the administration of what was considered to be a sufficient amount of UFH. In such cases, adjustments should be made with reference to multiple coagulation tests to prevent UFH overdose and bleeding complications, and VET can be a useful alternative POCT for monitoring anticoagulation with UFH. Further research is needed to determine which test best suits heparin monitoring. Whichever test is selected, it should be standardized to minimize the risk of error.

## Conclusions

ACT is not always reliable for estimating the effectiveness of UFH, especially in lower doses. As an alternative POCT to ACT, VET may be suitable for assessing low doses of UFH effectiveness with a heparinase assay, which provides specific measures of the heparin effect, such as CTR, and minimizes the variability in results as compared to ACT. Further evaluation of cost-effectiveness and optimal target range is needed to integrate VET into clinical practice for Impella management.

## References

[1] (2024). Abiomed Inc. Impella ventricular support systems for use during cardiogenic shock and high-risk PCI. https://www.accessdata.fda.gov/cdrh_docs/pdf14/p140003s018d.pdf.

[2] Maier CL, Sniecinski RM (2021). Anticoagulation monitoring for perioperative physicians. Anesthesiology.

[3] Elbadawi A, Elgendy IY, Mahmoud K (2019). Temporal trends and outcomes of mechanical complications in patients with acute myocardial infarction. JACC Cardiovasc Interv.

[4] Saito S, Shibasaki I, Matsuoka T (2022). Impella support as a bridge to heart surgery in patients with cardiogenic shock. Interact Cardiovasc Thorac Surg.

[5] Delmas C, Barbosa H, David CH (2023). Impella for the management of ventricular septal defect complicating acute myocardial infarction: a European multicenter registry. ASAIO J.

[6] Vandenbriele C, Arachchillage DJ, Frederiks P (2022). Anticoagulation for percutaneous ventricular assist device-supported cardiogenic shock: JACC review topic of the week. J Am Coll Cardiol.

[7] Faraoni D, DiNardo JA (2021). Viscoelastic hemostatic assays: update on technology and clinical applications. Am J Hematol.

[8] Allen TW, Winegar D, Viola F (2020). The Quantra® System and SEER sonorheometry. Trauma Induced Coagulopathy.

[9] McMichael AB, Ryerson LM, Ratano D, Fan E, Faraoni D, Annich GM (2022). 2021 ELSA adult and pediatric anticoagulation guidelines. ASAIO J.

[10] Leyvi G, Shore-Lesserson L, Harrington D, Vela-Cantos F, Hossain S (2001). An investigation of a new activated clotting time "MAX-ACT" in patients undergoing extracorporeal circulation. Anesth Analg.

[11] Jones PM, Bainbridge D, Dobkowski W, Harle CC, Murkin JM, Fernandes PS, Kiaii B (2011). Comparison of MAX-ACT and K-ACT values when using bivalirudin anticoagulation during minimally invasive hybrid off-pump coronary artery bypass graft surgery. J Cardiothorac Vasc Anesth.

